# Autophagy activation contributes to lipid accumulation in tubular epithelial cells during kidney fibrosis

**DOI:** 10.1038/s41420-018-0065-2

**Published:** 2018-06-27

**Authors:** Qi Yan, Yuan Song, Lu Zhang, Zhaowei Chen, Cheng Yang, Shan Liu, Xiaohan Yuan, Hongyu Gao, Guohua Ding, Huiming Wang

**Affiliations:** 1 Department of Nephrology, Renmin hospital of Wuhan University, Wuhan, China; 2 Department of Geriatrics, Tongji Hospital, Tongji Medical College, Huazhong University of Science and Technology, Wuhan, China; 3 Department of Nephrology, University Hospital of Hubei University for Nationalities, Enshi, China

## Abstract

Sustained activation of autophagy and lipid accumulation in tubular epithelial cells (TECs) are both associated with the kidney fibrosis progression. Autophagy has been found involved in the lipid metabolism regulation through a bi-directional mechanism of inducing lipolysis as well as promoting lipid droplet formation. However, whether and how autophagy influences lipid accumulation in kidney fibrosis remain unclear. In the current study, we show that UUO-induced lipid accumulation in tubular cells was significantly reduced when the pharmacological inhibitor 3-MA or CQ was administrated both in vivo and in vitro. Of interest, colocalization of LDs and autophagosomes, as well as colocalization of LDs and lysosomes were undetected in UUO-induced fibrotic kidneys, although lysosome function remained robust, indicating the lipid accumulation is lipophagy-lysosome pathway independent. TGF-β1-induced lipid droplets formation in HK-2 cells were decreased when the Beclin-1 expression was silenced, implying that autophagy-upregulated lipid droplets formation is Beclin-1 dependent. Finally, CQ treatment of UUO-induced fibrotic kidneys reduced the expression of α-SMA and tubular cell apoptosis and rescued the expression of E-cadherin, which was associated with the ameliorated lipid deposition. Therefore, our work documented that autophagy promotes lipid droplet formation in TECs in a Beclin-1-dependent manner, which causes renal lipotoxicity and contributes to the progression of kidney fibrosis.

## Introduction

Chronic kidney diseases (CKD), especially ESRD, impose a heavy socioeconomic burden on public health systems worldwide^1^. Kidney fibrosis is the major pathologic lesion and final pathway in end-stage CKD and is characterized by the accumulation of extracellular matrix (ECM), loss of the integrity of interstitial capillaries and infiltration of immune cells. The underlying mechanisms and fundamental pathways of kidney fibrosis are incompletely understood, the effective therapeutic methods or agents targeting kidney fibrosis are also lacking.

Macroautophagy (hereafter referred to as autophagy) is a fundamental stress response that participates in maintaining cellular hemostasis. Autophagy begins with the formation of double-membrane-bound autophagic vesicles (autophagosomes) that subsequently engulf recycled proteins and damaged organelles and eventually fuse with lysosomes. Autophagy can be activated by multiple factors, including nutrient starvation, energy deprivation or pathogen infection, and has been implicated in the pathogenesis of various diseases such as carcinoma, neurodegenerative diseases, liver diseases, cardiovascular diseases, as well as the kidney diseases^2–4^. The cytoprotective role of autophagy in diabetic nephropathy (DN) and acute kidney injury (AKI) has been well documented^5–7^. It has been found that autophagy can be induced in TECs either in vivo of UUO fibrotic model or in vitro with TGF-β1 stimulation^8–14^, while the precise role and detailed mechanism of autophagy on kidney fibrosis remains elusive. Recently, Livingston et al^15^ demonstrated that UUO initiated persistent autophagy in TECs and exerts profibrotic effects in kidney fibrosis.

Altered lipid metabolism and excessive lipid deposition in the kidneys have been designated as an important detrimental factor in the kidney diseases progression^16^. The so-called lipotoxicity due to the ectopically lipids accumulation has also been observed in podocytes and TECs that causing cell injury and dysfunction, especially in the context of AKI and DN^17–19^. Cytosolic lipid homeostasis is tightly regulated and depends on the balance between lipid intake and lipid degradation, in which multiple factors or mechanisms are involved. Autophagy has been reported to play an essential role in regulating intracellular lipid metabolism^20^. Autophagy may regulate lipid metabolism in dual-direction^20–23^, on one side, autophagy induces lysosomal lipolysis from lipid droplets, known as lipophagy^20^, and on the other side, it is involved in the formation of lipid droplets (LDs)^21, 24^.

The sustained activation of autophagy^15^ and the excessive lipid accumulation^19, 25–27^ in TECs of fibrotic kidney have been described separately. However, whether autophagy and lipid deposition are interrelated in the kidney fibrosis is still unclear. In the present study, we investigated the effects of autophagy activation on the lipid accumulation in TECs both in UUO-induced fibrotic kidney and in vitro-cultured HK-2 cells, and elucidated the underlying mechanism of autophagy on lipid metabolism during kidney fibrosis.

## Results

### Autophagy activation in fibrotic kidneys was closely associated with lipid accumulation

**Fig. 1:**
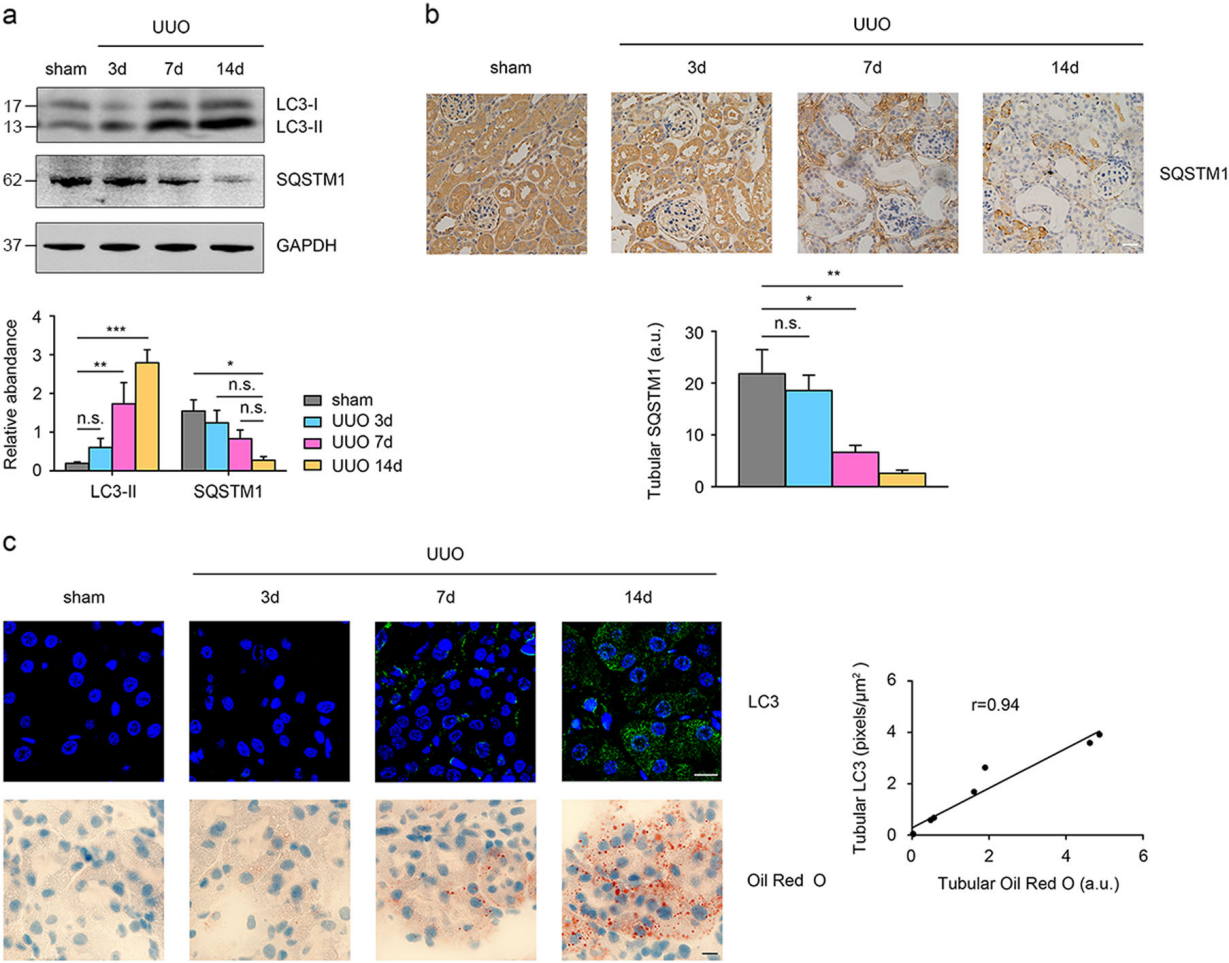
Correlation between autophagy activation and lipid accumulation in fibrotic kidneys. C57BL6 mice were subjected to either sham operation or UUO treatment at the indicated time points (3, 7 and 14 days). **a** Kidney samples were collected for western blotting staining with LC3, SQSTM1 and GAPDH antibodies. GAPDH sets as loading control. Schematic representation of band intensity of indicated proteins. The data are calculated from three independent experiments and are expressed as the mean ± SEM. *P < 0.05; **P < 0.01; ***P < 0.001. **b** Kidney samples were collected for IHC staining with anti-SQSTM1 antibody. Representative images from three independent experiments are shown above. Scale Bar: 50 μm. Schematic representation of SQSTM1 positive staining. The data are expressed as the mean ± SEM. *P < 0.05; **/*P* < 0.01. **c** Kidney samples were collected for immunofluorescence staining with anti-LC3 antibody and Oil Red O staining. Representative images from three independent experiments are shown above. Scale bar: 10 μm. Schematic representation of the correlation between level of tubular Oil Red O staining and tubular LC3 expression (*n* = 8). n.s. no significance

Previous studies have indicated that autophagy is induced in TECs in fibrotic kidney mouse models such as UUO^12, 15, 28^ or tubule-specific overexpression TGF-β1 genetic mice^14^, and considerable lipid accumulation in TECs was detected in folic acid-induced fibrotic kidneys^19^, but whether these two events are interrelated in kidney fibrosis remains unknown. We first examined the autophagic activity during different stages of kidney fibrosis. As shown in Fig.1a, expression of LC3-II was gradually elevated in a time-dependent manner in fibrotic kidneys; whereas SQSTM1, a selective autophagy substrate during autophagy activation, was gradually degraded during UUO duration in a time-dependent manner, as detected by both western blotting (Fig. 1a) and IHC staining (Fig. 1b). These results indicated that autophagy was induced in kidney following UUO initiation.

We then studied the relationship between autophagy activation and lipid deposition during kidney fibrosis. The expression of the autophagosome marker LC3 was detected using confocal fluorescence microscopy in the obstructed kidneys of mice at 3, 7 and 14 days after UUO treatment. As shown in Fig. 1c, few LC3-positive puncta were observed in sham-operated kidneys, while in the UUO kidney, the LC3 puncta gradually accumulated in a time-dependent manner. Oil Red O staining displayed that neutral lipids accumulation in TECs was also increased in time-dependent manner and is concomitant with the alteration of LC3 puncta accumulation. The positive association between the intensity of LC3 puncta accumulation and lipid accumulation was further confirmed by Pearson’s correlation analysis.

### Pharmacological inhibition of autophagy by 3-MA or CQ reduced lipid accumulation in the TECs of UUO mouse model

**Fig. 2:**
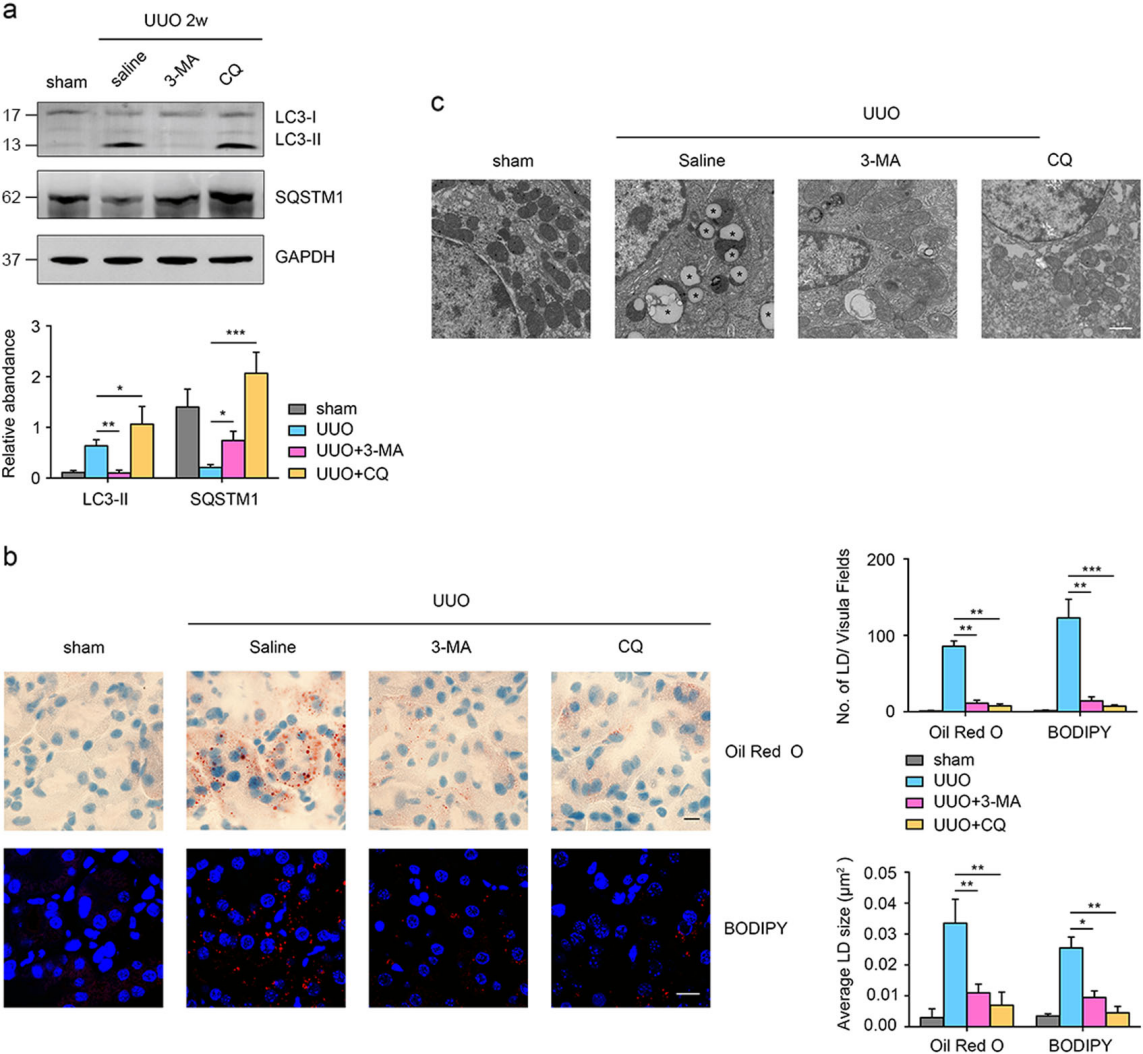
Pharmacological inhibition of autophagy by 3-MA or CQ reduces TECs lipid accumulation of fibrotic kidneys after UUO. Mice were divided into four groups: sham operation, UUO surgery, UUO surgery + 3-MA (30 mg/kg/d) and UUO surgery + CQ (60 mg/kg/d). Mice were killed on day 14, and kidney samples were collected for further experiments. **a** Western blotting analysis of the expression of LC3, SQSTM1, and GAPDH. GAPDH was set as a loading control. A schematic representation of band intensity of the indicated proteins. The data are calculated from three independent experiments and are expressed as the mean ± SEM. *P < 0.05; **P < 0.01; ***P < 0.001. **b** Representative images of Oil Red O staining and BODIPY 558/564 staining from three independent experiments are shown above. Scale bar: 10 μm. Schematic representation of the number of LDs and average LDs size form both Oil Red O and BODIPY staining. The data are expressed as the mean ± SEM. *P < 0.05; **P < 0.01; ***P < 0.001. **c** Representative electron micrographs in TECs from indicated groups are shown. LDs are round membrane-coated organelles filled with high electron-dense inert lipids. Scale bar: 1 μm

To investigate the potential role of autophagy in regulating lipid metabolism in fibrotic kidneys, we examined the effects of two pharmacological autophagy inhibitors, 3-MA and CQ, on lipid metabolism during UUO-induced kidney fibrosis. CQ can block lysosomal degradation of autophagosomes, and thus cause accumulation of LC3-II and SQSTM1. 3-MA will block autophagosome formation through selectively PI3K pathway inhibition, resulting in the reduction of LC3-II expression and SQSTM1 accumulation. We studied the efficiency of 3-MA and CQ in autophagy inhibition during kidney fibrosis. As shown in Fig. 2a, UUO-induced autophagy activation was efficiently mitigated by the administration of 3-MA and CQ, as indicated by the altered expression pattern of LC3-II and SQSTM1, respectively. Similar to the autophagy alteration, 3-MA and CQ treatment also caused the dramatically reduction in lipid accumulation, which was detected by Oil Red O and BODIPY 558/568 (Red C12) staining (Fig. 2b). We also performed electron microscopy to directly observe the ultrastructure of TECs. Electron micrographs revealed the extensive accumulation of intracellular LDs in TECs from the kidneys of mice in the UUO group, showing around membrane-coated organelles filled with high electron-dense inert lipids. In contrast, the LDs in the UUO + 3-MA group or UUO + CQ group were notably decreased. Surprisingly, no LDs were detected in the kidneys of sham-operated mice (Fig. 2c).

**Fig. 3:**
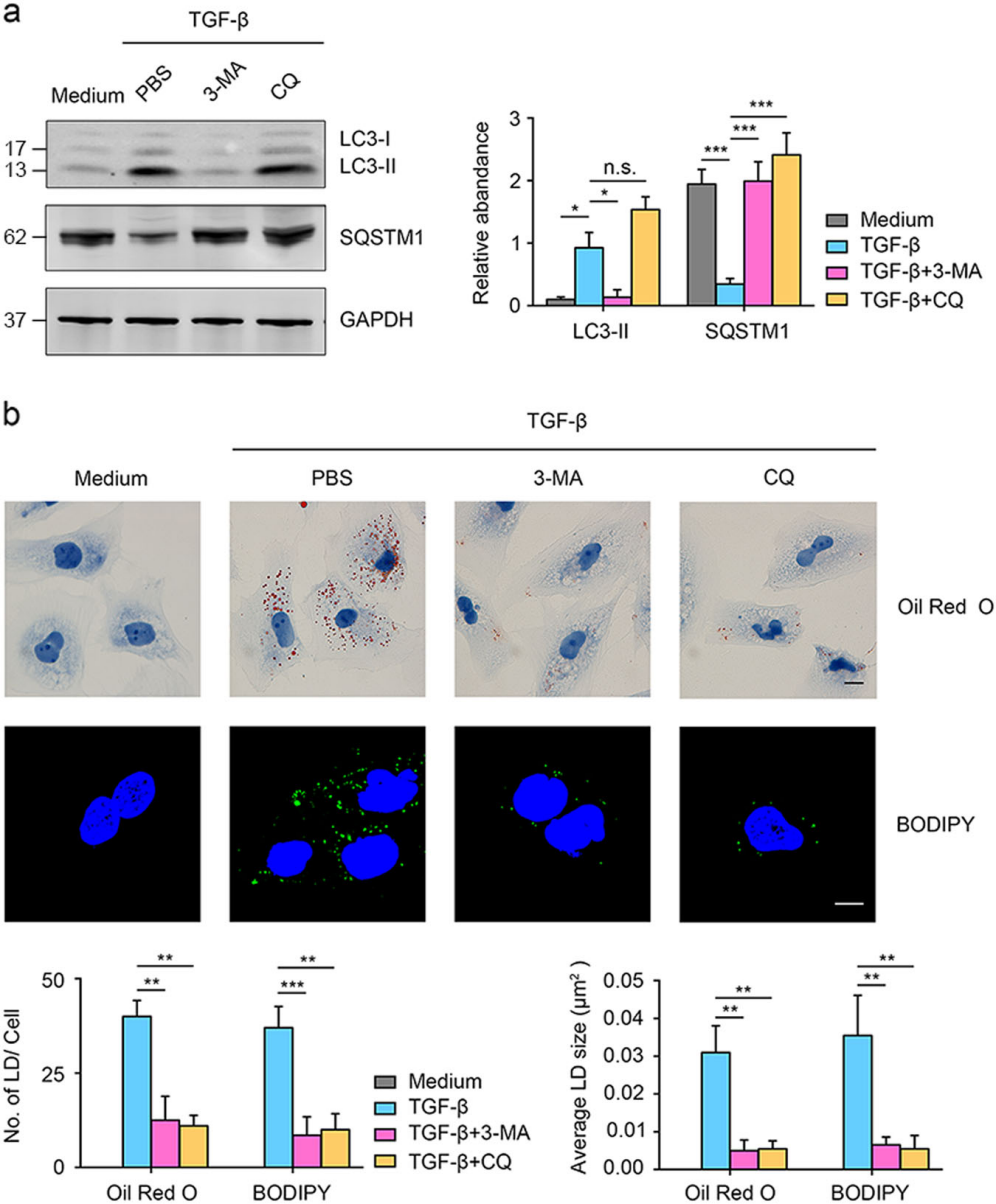
Pharmacological inhibition of autophagy by 3-MA or CQ reduces lipid deposition in TGF-β1-induced tubular cells. Cells were divided into four groups: medium, TGF-β1 + PBS, TGF-β1 + 3-MA (10 mM) and TGF-β1 + CQ (20 μM). **a** Western blotting analysis of the expression of LC3, SQSTM1 and GAPDH. GAPDH set as loading control. Schematic representation of band intensity of the indicated proteins. The data are calculated from three independent experiments and are expressed as the mean ± SEM. *P < 0.05; ***P < 0.001. **b** Cells were analyzed using Oil Red O staining and BODIPY staining, and one representative image from three independent experiments is shown. Scale bar: 20 μm. Schematic representation of the number and average size of LDs from both Oil Red O staining and BODIPY staining. The data are expressed as the means ± SEM. **P < 0.01; ***P < 0.001; n.s. no significance

The effects of autophagy on lipid accumulation were further studied in vitro in cultured HK-2 cells. As shown in Fig. 3a, TGF-β1 led to autophagy induction in HK-2 cells, which was further suppressed by both 3-MA and CQ treatment, as suggested by the changes of expression of LC3-II and SQSTM1 by western blotting. Both autophagy activation and lipid accumulation were triggered by the TGF-β1 stimulation, which are consistent with previous reports^8, 14, 19^. 3-MA or CQ treatment of HK-2 cells remarkably suppressed not only the TGF-β1-initiated autophagy activation but also the LDs accumulation in HK-2 cells (Fig. 3b). These concordant results from in vivo and in vitro studies strongly suggest that lipid accumulation in TECs of UUO fibrotic kidney is at least partly promoted by autophagy activation.

### The lipid accumulation in UUO fibrotic kidney was independent of the lipophagy-lysosome system

**Fig. 4:**
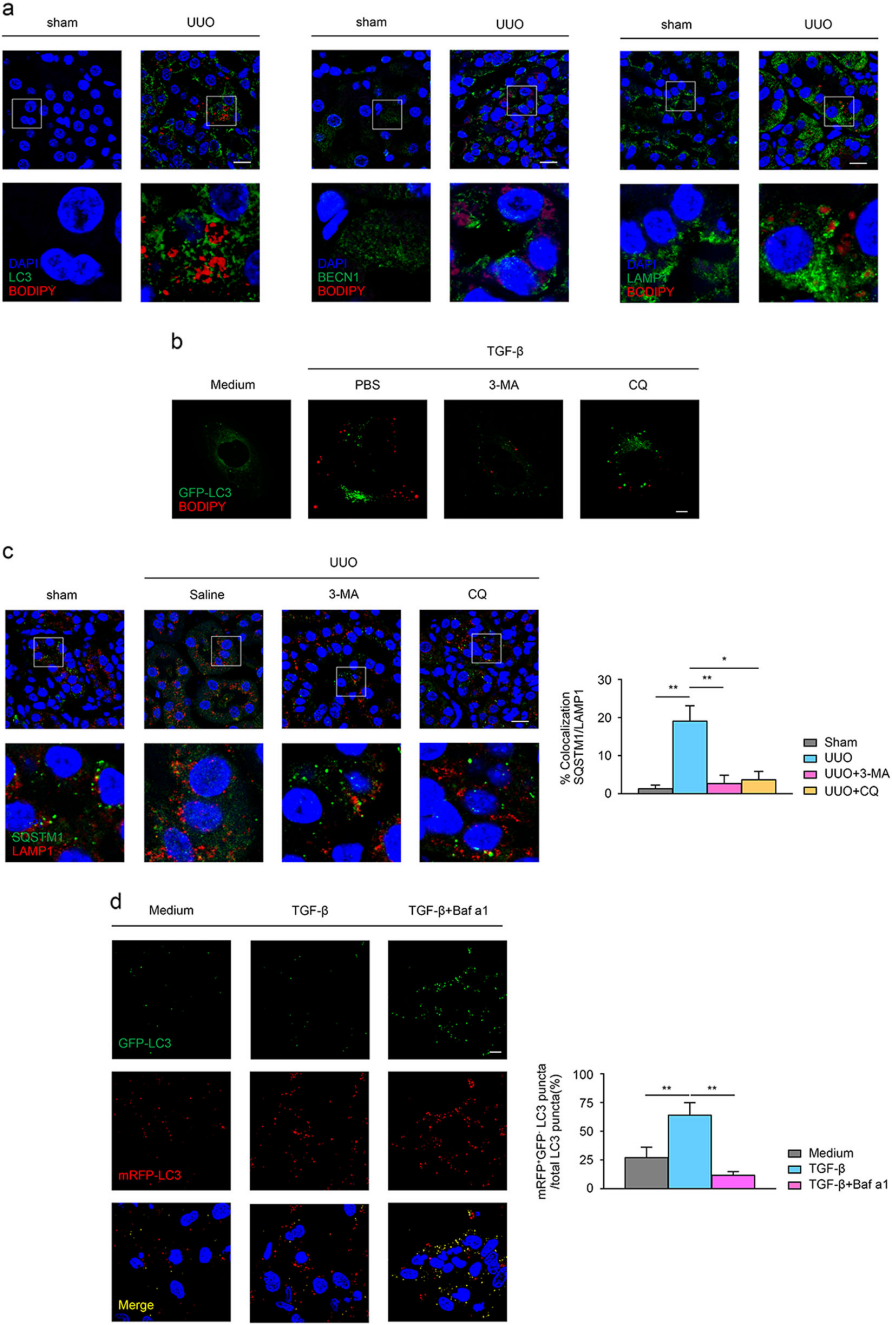
Lipid metabolism in kidney fibrosis induced by UUO is independent of the lipophagy-lysosome system. **a** Kidney samples were collected for immunofluorescence staining. Representative images of coimmunolabeling for LC3 & BODIPY 558/564, Beclin-1 & BODIPY 558/564 and LAMP1 & BODIPY 558/564 from three independent experiments are shown above. Scale bar: 10 μm. **b** Cultured HK-2 cells were transfected with the GFP-LC3 plasmid and treated as same as in Fig. 3. Representative images of coimmunolabeling for LC3 & BODIPY 493/503 from three independent experiments are shown above. Scale bar: 10 μm. **c** Mice treatment was as same as described in Fig. 2. Representative images of coimmunolabeling for LAMP1 and SQSTM1 from three independent experiments are shown above. Scale bar: 10 μm. Schematic representation of the colocalization between the Red and Green channels (*n* = 3). The data are expressed as the means ± SEM. *P < 0.05. **P < 0.01. **d** Cultured HK-2 cells were transfected with the tfLC3 plasmid for 48 h and subsequently treated with TGF-β1 (50 ng/ml) in the absence or presence of Baf a1 (10 nM) for 24 h. The cells were subsequently analyzed using confocal microscopy to detect the fluorescence, and one representative image from three independent experiments is shown. Scale bar: 5 μm. Schematic representation of the number of mRFP^+^GFP^−^ LC3 puncta per total LC3 puncta. The Data are expressed as the means ± SEM. **P < 0.01

Lipophagy is an essential mechanism for intracellular lipid degradation, through which lipids are sequestrated into autophagosomes and further fused with lysosomes^20^. One feature of lipophagy is the colocalization between autophaogosomal marker (such as LC3 and Beclin-1) and LDs (marked as BODIPY). Above results demonstrated that autophagy in UUO fibrotic kidney was accompanied with no lipid depletion but rather lipid accumulation, implying the sustained autophagy has not resulted in the enhanced lipid degradation. Thus, we checked the status of autophagy-related lipolysis in UUO fibrotic kidney by examining the colocalization between autophagosome and LDs. As displayed in Fig. 4a, colocalization between LC3 and BODIPY, BEECN1 and BODIPY, as well as LAMP and BODIPY was not detected in the kidneys of either sham-operated mice or UUO mice. Moreover, we check up the results of transmission electron microscope and found that in UUO-induced fibrotic kidneys, no colocalization between LDs and double-membrane autophagosomes or lysosomes were found (data not shown). The LC3 and BODIPY colocalization was also not detected in the GFP-LC3-transfected HK-2 cells under TGF-β1 stimulation regardless the autophagy inhibition by 3-MA or CQ (Fig. 4b). In addition, investigated the autophagosome formation in the UUO fibrotic kidney, since the autolysosome is critical in the autophagy-mediated lipid degradation. As shown in Fig. 4c, SQSTM1, a substrate for autophagic degradation, was colocalized with LAMP1 in UUO-treated kidneys, and this colocalization was diminished when 3-MA or CQ was administrated. Furthermore, we utilized the tfLC3 plasmid, in which mRFP is pH-insensitive and stably expressed, but GFP can be quenched in the acidic lysosomal environment to check up the lytic function of autolysosome. As shown in Fig. 4d, mRFP^+^GFP^-^ LC3 puncta were detected in the cells in normal conditions, indicating a basal autophagy-lysosome activity. Upon TGF-β1 stimulation, mRFP^+^GFP^−^ LC3 puncta increased significantly. Moreover, to set a positive control in the test, the cells were treated with TGF-β1 in the combination with Baf a1, which is an autophagy inhibitor that prevents the fusion of autophagosomes with lysosomes; immunofluorescence revealed a significant increase in intracellular mRFP^+^GFP^+^ LC3 puncta, which means that quenching of GFP^+^ LC3 in autolysosome can be inhibited. These results suggested that UUO-induced autophagy in TECs is functional normal from the upstream process of autophagy initiation to the downstream autolysosome formation, the accumulation of lipid in cell should be ascribed to the failure of LDs being sequestered to the autophagosome.

### Beclin-1 was involved in the autophagy-induced LDs accumulation in TECs of fibrotic kidney

**Fig. 5:**
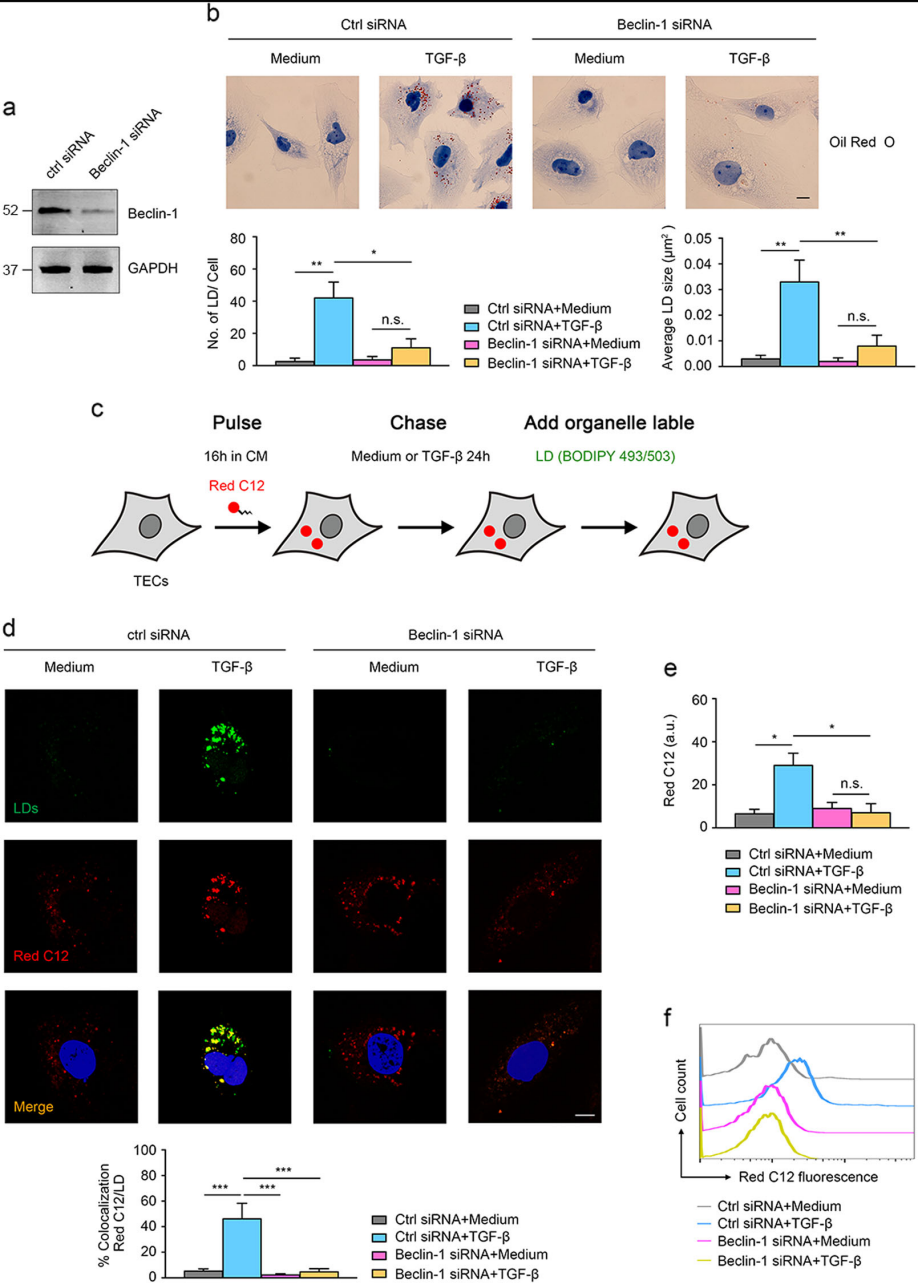
Beclin-1 is involved in LDs formation in TGF-β treated tubular cells. **a** HK-2 cells were transfected with scramble siRNA or Beclin-1 siRNA for 72 h. Western blotting analyses revealing the downregulation of Beclin-1 in HK-2 cells after Beclin-1 siRNA transfection. **b** Cells were analyzed using Oil Red O staining and one representative image from three independent experiments is shown. Scale bar: 20 μm. Schematic representation of the number and average size of LDs from Oil Red O staining. The data are expressed as the means ± SEM. *P < 0.05; **P < 0.01. **c** Schematic illustration of fluorescence Red C12 pulse-chase assay. **d** Cells were treated as described before and stained with the LDs marker BODIPY 493/503. Confocal microscopy was used to acquire the fluorescence images. One representative image from three independent experiments is shown. Scale bar: 5 μm. Schematic representation of the colocalization between Red C12 and LDs (*n* = 3). The data are expressed as the means ± SEM. ***P < 0.001. **e** Schematic representation of the staining of fluorescence Red C12 from Fig. 5d. The data are expressed as the means ± SEM. *P < 0.05. **f** Cells were analyzed using flow cytometry to detected intracellular Red C12 fluorescence. One representative image from three independent experiments is shown. n.s. no significance

Autophagy-related genes (ATGs) have been reported involved in LDs formation^21, 23, 24, 29^. Among these candidate ATGs, Atg6 (mammalian homolog also called Beclin-1) is relatively unique in its not being “autophagy specific”. Although the function of Beclin-1 in autophagy has been well-established, as ER-localized Beclin-1 is interacted with Atg14 and Vps34 and play a critical role in early autophagosomal biogenesis^30^. Recently, studies also found that Beclin-1 protein is part of lipid kinase complex and closely related to cytosolic lipid metabolism^23, 31^. Previous studies demonstrated that loss of Beclin-1 resulted in the decline of lipid storage in cells^23^, while overexpression of Beclin-1 in a mouse model of Niemann–Pick disease led to the upregulation and aberrant lipid accumulation in organs and cells^32^. We speculated that Beclin-1 is responsible for the autophagy-induced lipid accumulation in TECs of fibrotic kidney. To test this speculation, the lipid accumulation in *Beclin-1* knockdown HK-2 cells upon TGF-β1 stimulation was observed. Figure 5a displays that Beclin-1 was efficiently knocked down in HK-2 cells with *Beclin-1* siRNA transfection. The lipid level, as presented by Oil Red O staining, was increased in Ctrl siRNA-transfected HK-2 cells in response to TGF-β1 stimulation, but not significant in *Beclin-1* knockdown HK-2 cells (Fig. 5b).

A pulse-chase assay with BODIPY 558/568 C12 (Red C12) was further performed to visualize the formation of LDs in HK-2 cells (Fig. 5c). As shown in Fig. 5d, the Red C12 signal was preserved in neutral lipids and showed no colocalization with the LDs marker BODIPY 493/503 in cells under normal condition. TGF-β1 induced a considerable increase of LDs and significant colocalization between Red C12 and BODIPY 493/503 (Fig. 5d), suggesting that TGF-β1 treatment caused redistribution of fatty acids to LDs. However, *Beclin-1* knockdown in HK-2 cells led to a dramatic decrease in the average size of LDs and the colocalization between the Red C12 signal and BODIPY 493/503 in response to TGF-β1 treatment. We also observed that the TGF- β1-upregulated Red C12 fluorescence staining were dramatically reduced in *Beclin-1*-knockdown HK-2 cells (Fig. 5e), this was further confirmed by flow cytometry analyzing the intracellular intensity of Red C12(Fig. 5f), indicating that defective LDs formation in *Beclin-1*-knockdown cells might be due to impaired influx of intracellular free fatty acids (FAs).

The above results suggested that autophagy-induced lipid accumulation in TECs of fibrotic kidney was dependent on Beclin-1. It drove us to examine the expression level of Beclin-1 in TECs of fibrotic kidney. Supplementary Fig. S1 showed that the expression of Beclin-1 was significantly increased in UUO-induced fibrotic kidneys compared with sham-operated group, similar results were found in TGF-β1-treated HK-2 cells. This indicates that Beclin-1 expression is upregulated in TECs in fibrotic kidney, and which might mediate the excessive lipid deposition in fibrotic kidney.

### Pharmacological inhibition of autophagy by CQ ameliorated UUO-induced fibrotic lesions concomitantly with declined lipid deposition

**Fig. 6:**
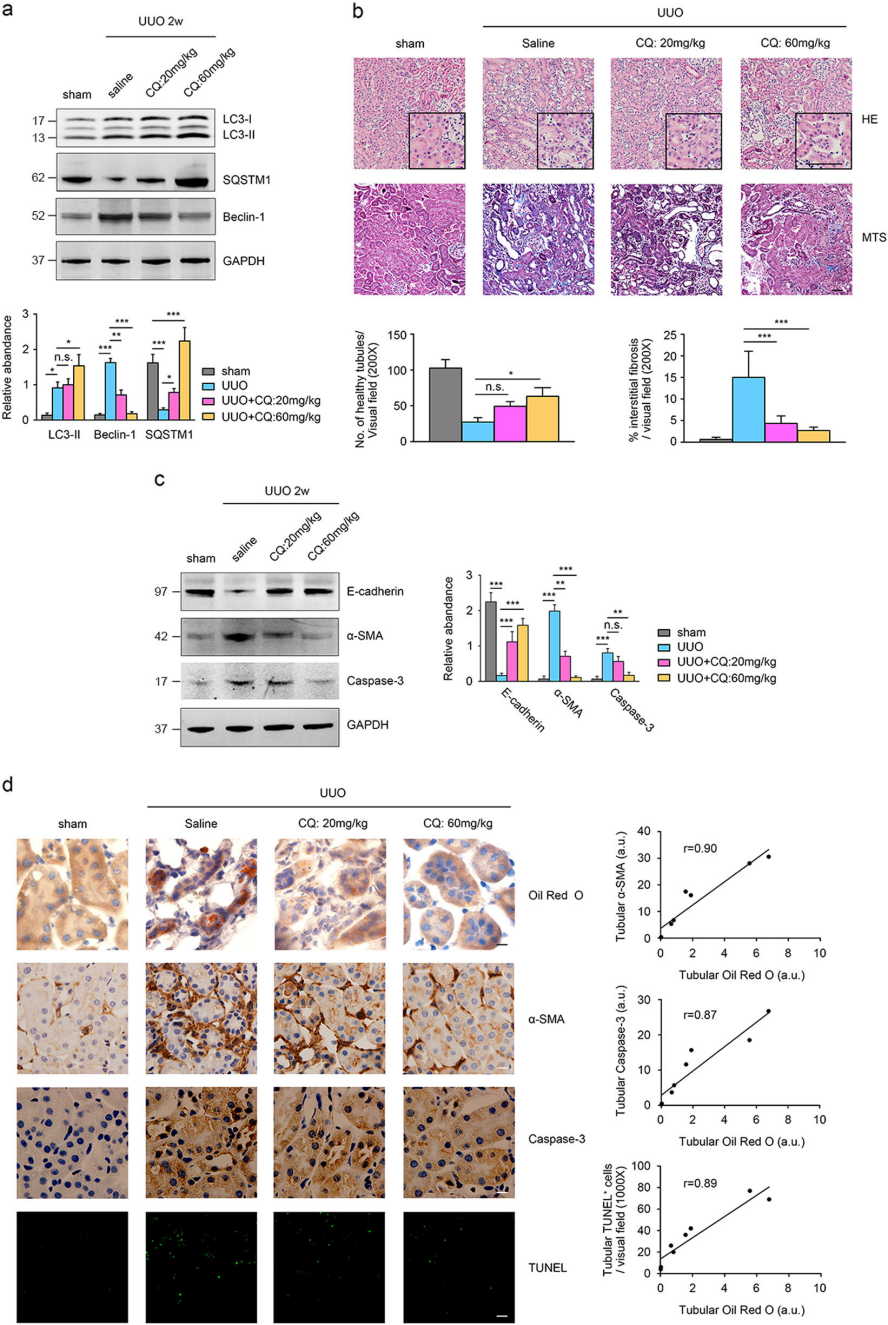
Pharmacological inhibition of autophagy by CQ ameliorates fibrotic responses consistent with decreases in lipid deposition induced by UUO. The mice were divided into the following four groups: sham operation, UUO surgery + Saline, UUO surgery + CQ (20 mg/kg/d) and UUO surgery + CQ (60 mg/kg/d). The mice were killed on day 14, and the kidney samples were collected for further experiments. **a** Kidney samples were collected for western blotting staining with LC3, Beclin-1, SQSTM1 and GAPDH antibodies. GAPDH sets as loading control. Schematic representation indicates band intensity of indicated proteins. The data are calculated from three independent experiments and are expressed as the mean ± SEM. *P < 0.05; ***P < 0.001. **b** Representative images of HE and MTS staining from three independent experiments are shown above. Scale bar: 25 μm. Schematic representation of number of health tubules based on HE and % of interstitial fibrosis based on MTS staining (*n* = 3). The data are expressed as the means ± SEM. n.s. means no statistical significance; *P < 0.05; ***P < 0.001. **c** Kidney samples were collected for western blotting staining with E-cadhein, α-SMA, caspase-3 and GAPDH antibodies. GAPDH sets as loading control. Schematic representation indicates band intensity of the indicated proteins. The data are calculated from three independent experiments and are expressed as the mean ± SEM. **P < 0.01; ***P < 0.001. **d** Representative images of Oil Red O staining, IHC staining for α-SMA, cleaved caspase-3 and tubular TUNEL staining from three independent experiments are shown above. Scale bar: 10 μm. Schematic representation of the correlation between level of tubular Oil Red O staining and expression of α-SMA, expression of cleaved caspase-3 and TUNEL-positive cells, respectively (*n* = 8). n.s. no significance

Lipotoxicity due to lipid accumulation in the kidneys plays a key role in the fibrosis progression^17, 33^. On the basis of previous findings of this study, that UUO-initiated autophagy contributed to the lipid accumulation, it is rational to raise the possibility of protecting kidney from lipotoxicity by autophagy inhibition. Indeed, administration with CQ on UUO mice was effective in inhibiting autophagy activation, which was manifested by the LC3-II and SQSTM1 expression; additionally, CQ treatment also significantly reduced the expression of Beclin-1 in UUO kidney, indicating that one possible mechanism of chloroquine (CQ) reducing the lipid accumulation in TECs during renal fibrosis is by inhibiting Beclin-1 expression (Fig. 6a). Meanwhile, HE and MTS staining revealed that tissue damage was also mitigated in UUO kidney receiving CQ treatment in a dose-dependent manner (Fig. 6b). Western blotting analysis for E-cadherin, α-SMA and caspase-3 were performed and the results demonstrated that the expression of α-SMA and cleaved caspase-3 were both significantly attenuated in a dose-dependent manner under CQ treatment, whereas the expression of E-cadherin was significantly reduced in UUO kidney but increased after CQ treatment, indicating that UUO-induced renal damage were ameliorated after CQ engagement (Fig. 6c).

Lipid metabolism in UUO kidney with the CQ administration was also evaluated. Oil Red O staining of kidney tissues revealed that lipid accumulation was remarkably decreased in CQ-treated kidneys in a dose-dependent manner. The expression of α-SMA and cleaved caspase-3, and the tubular apoptotic cells were all significantly attenuated in a dose-dependent manner under CQ treatment (Fig. 6d). Moreover, the correlation analysis revealed that lipid accumulation in kidney was positively related to the α-SMA expression (*r* = 0.89), cleaved caspase-3 expression (*r* = 0.90), and tubular apoptotic cells (*r* = 0.90) (Fig. 6d). Since LDs formation has been recognized as a marker of the increased lipid accumulation and ensued lipotoxicity in renal diseases^33–35^, these results suggest that autophagy inhibition by CQ is effective in preventing against the lipotoxicity and tissue damage in UUO fibrotic kidney.

## Discussion

**Fig. 7:**
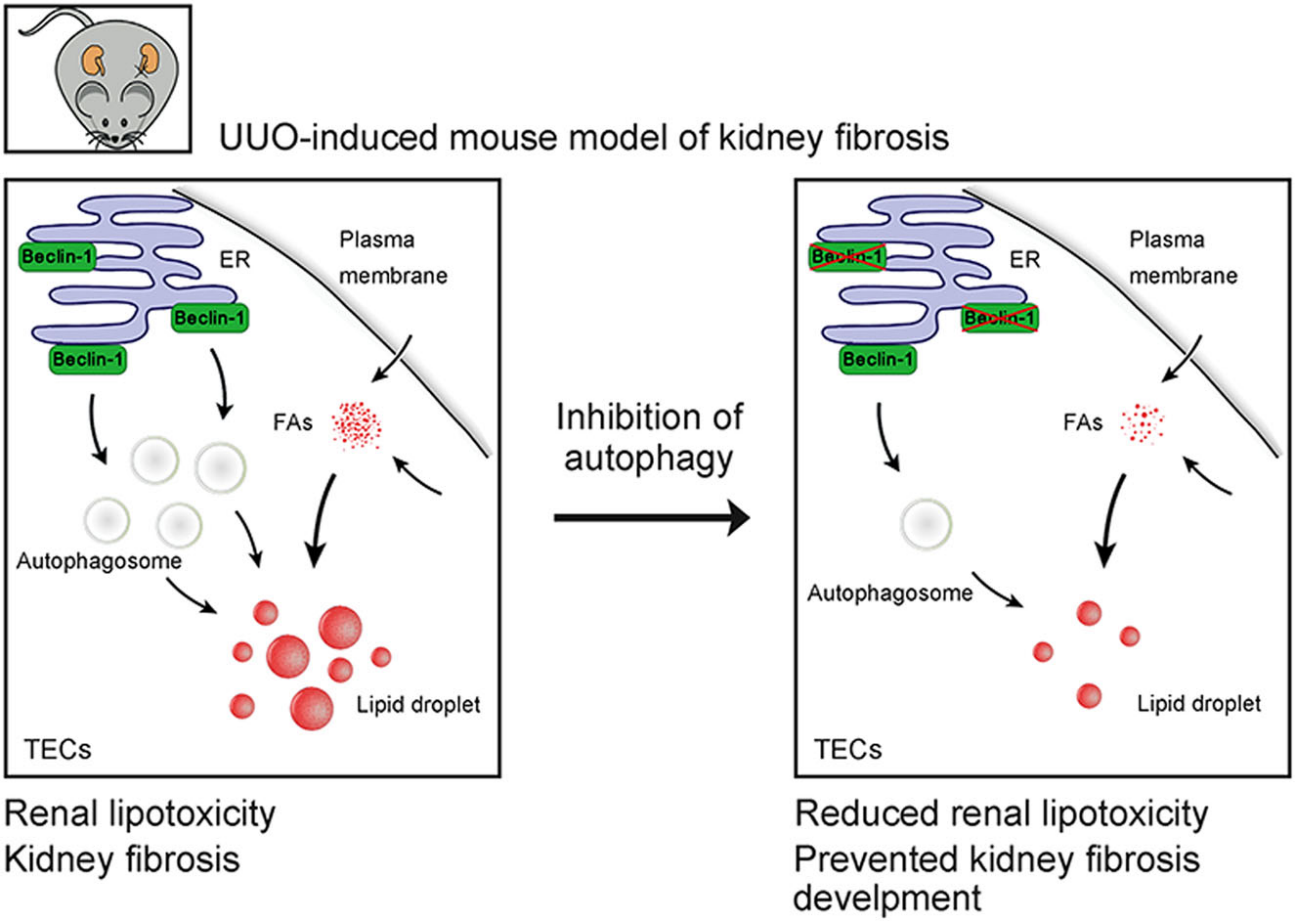
Beclin-1 dependent autophagy activation contributes to lipotoxicity and fibrotic responses induced by UUO. In TECs from UUO-induced fibrotic kidneys, Beclin-1 is recruited to endoplasmic reticulum (ER) and initiates the biogenesis of autophagosome, which might facilitate the lipid droplets (LDs) formation induced by redundant intracellular Fatty acids (FAs), causing renal lipotoxicity and fibrotic responses; Inhibition of autophagy reduces the expression of Beclin-1, which impairs the intensity of intracellular free FAs and decreases the ectopic deposition of LDs in TECs, leading to reduction of renal lipotoxicity and prevention of the progression of kidney fibrosis

Persistent activation of autophagy and the abnormality of lipid metabolism both are prominent features associated with renal fibrosis progression. Although their roles of causing kidney lesions have been fully described, the interrelationship between these two events in kidney fibrosis remains unclear. In the present study, we found that UUO-induced autophagy in TECs played critical role in the lipid accumulation and the lipotoxicity in the kidney fibrosis progression, the upregulation of autophagy in fibrotic TECs did not lead to the increasing of lipophagy but rather contributed to the Beclin-1-dependent cytosolic lipid accumulation. Inhibition of autophagy by the administration of CQ was effectively in ameliorating the fibrotic tissue damage as well as the kidney lipid deposition in UUO mouse model (Fig. 7). For the first time, we elucidated a novel mechanism of autophagy in kidney fibrosis by mediating the lipid metabolism in tubular cells, which might extend our current knowledge about the detailed mechanisms of autophagy in kidney fibrosis and would shed light on the way of seeking potential therapeutic strategies targeting kidney fibrosis.

TECs are metabolically active cells with high energy demands. TECs primarily use fatty acids as fuel, and the mitochondrial β-oxidation of fatty acids is a major energy source for ATP generation, whereas glucose catabolism is less demanded and is not a key contributor to energy production in TECs^19, 36^. Unlike adipocytes, which have massive intracellular LDs storage under physiological conditions, TECs harbor much little number of LDs to maintain its energy homeostasis^37^. Here we also found that the constitute number of LDs in TECs either in vivo or in vitro was at very low level. Lipid overload in TECs, which would be a result of excessive dietary intake or dysfunction in lipid consumption or degradation, exerts vicious effects termed lipotoxicity through the generation of reactive oxygen species, release of proinflammatory and profibrotic factors, as well as cell apoptosis^33, 38, 39^. This has been observed in obesity-associated kidney diseases, diabetic nephropathy, and kidney fibrosis progression^16, 17, 19, 33^. The excess ectopic accumulation of lipid in TECs could be a subsequence of systemic dyslipidemia, but sometimes, it is an outcome of the imbalance of cellular lipid metabolism, because renal lipid accumulation occurred independent of the systemic lipid disorder in the contexts of hypertensive nephrosclerosis, focal segmental glomerulosclerosis (FSGS), or minimal change disease (MCD)^33^. Particularly, it has been manifested that this has it occurence in lower expression of key enzymes and regulators of fatty acid oxidation (FAO) and in higher intracellular lipid deposition in humans and mouse models with tubulointerstitial fibrosis^19^. Our findings here demonstrated that persistent autophagy activation in TECs is another essential mechanism underlying the cellular lipid accumulation and lipotoxicity in kidney fibrosis.

Autophagy was initially found to play a role of lipophagy, through which cellular lipids stored in LDs are sequestered to autophagosomes for degradation as an energy source^20^. Lipophagy has been identified as an important mechanism of energy supporting in hepatocytes, adipocytes^40, 41^,macrophages^42^, fibroblasts^43^ as well as neurons^44^. Recently, Minami, S, et. al proved that prolonged starvation can trigger lipophagy in TECs to combat cellular energy depletion^45^. However, knowledge of whether autophagy contribute to the lipid abnormality in fibrotic kidney is still elusive. Considering the facts that the coexistence of persistent autophagy activation and excess lipid accumulation in TECs of the fibrotic kidney, and previous findings that autophagy may promotes lipid droplet formation^21, 24^, we speculated that the excess lipid deposition in fibrotic kidney might partly be ascribed to autophagy activation in TECs. Actually, in this study, we found that the concomitant events of autophagy and lipid accumulation in TECs in fibrotic kidney are not occurred in parallel but is being closely interrelated; autophagy activation is a critical cause in the lipid deposition and lipotoxicity during kidney fibrosis. It is intriguing that autophagy in TECs-induced by starvation leads to lipophagy and to elicit the cytoprotective effects^45^, whereas the UUO-initiated autophagy in TECs exerts the vicious effects of lipocytoxicity. We presume that autophagy is a cellular response with multifunction to cope with different challenges, crisis, or other certain status, but the underlying mechanism needs more studies.

Autophagy-related genes (ATGs) have been reported involved in LDs formation^21, 23, 24, 29^. Some components of autophagy have been identified closely related to the intracellular LDs formation. For instance, MAP1-LC3 conjugation system is required for intracellular LDs formation in hepatocytes^21^. ATGs are involved in the formation of LDs in both *C. elegans*^23^ and cultured mammalian cells^29^. Takagi et al^24^ found that ATG5 plays a crucial role in lipid metabolism by regulating LDs formation in the kidneys. Beclin-1 (Atg6) is relatively distinct and is different from other ATGs for its manifestation of “non-autophagy-specific” role. Beclin-1 protein is part of lipid kinase complex and closely related to cytosolic lipid metabolism^23, 31^. Previous studies suggested that Beclin-1 possess the potential of promoting cellular lipid storage^23, 32, 46, 47^. Our study here also demonstrated that Beclin-1 was responsible for the autophagy-induced lipid accumulation in TECs in fibrotic kidney. These findings indicate that there is a division of labor between the ATGs, through which mechanism the autophagy pathway is redirected to elicit the effect of lipid degradation or accumulation.

## Materials and Methods

### Animals

Male C57BL6 mice (8–10 weeks old) were used in these experiments. The animals were maintained in a specific pathogen-free (SPF) environment in the laboratory animal center of Wuhan University. All mice handling and experimentation was conducted according to the guidelines of the National Health and Medical Research Council of China. All mice protocols were approved by the animal ethics review board of Wuhan University.

Mice were fed ad lib with normal diet during whole experimental period. For the UUO model, the mice were first anesthetized with 2% isoflurane (i.p.), and subsequently the left ureter was exposed and isolated following mid-abdominal incision. For UUO, the mid-ureter was obstructed using 2-point ligations with silk sutures(4–0), and sham-operated mice underwent the same procedure without obstruction of the left ureter. After surgery, the incision was closed, and the mice were bred for indicated time points prior to euthanization. The kidney tissues were collected for further experiments.

For the pharmacological inhibition of autophagy treatment, the mice received 3-MA (30 mg/kg/d) or CQ (20 or 60 mg/kg/d) injection (i.p.) at 1 h prior to surgery and continuously received CQ daily for the UUO duration. The mice were killed on day 7 after surgery, and the kidneys were obtained for further analysis.

### Cell culture

The human tubular cell line HK-2 (purchased from China Centre for Type Culture Collection) was cultured in DMEM/F12 medium supplemented with 10% fetal bovine serum (FBS) and 1% penicillin/streptomycin (all from Life Technologies, Carlsbad, CA)

Cells were treated or not with 50 ng/ml of TGF-β1 in serum-free DMEM/F12 medium for 24 h. For some experiments, the cells were also incubated with TGF-β1 plus 10 mM 3-MA, 20 μM CQ or 10 nM Baf a1 in serum-free medium for 24 h. For gene disruption, the cells were treated with BECN-1 siRNA using HiPerFect transfection (both from Qiagen, German) according to the manufacturer’s instructions. The efficiency of transfection was assessed based on protein expression. The tfLC3 plasmid and GFP-LC3 plasmid transfection was performed using X-tremeGENE HP DNA transfection reagent (Roche Diagnostics GmbH, Mannheim, Germany) according to the manufacturer’s instructions. The tfLC3 (21074) plasmid and GFP-LC3 plasmid (22405) was both obtained from Addgene.

### **Rea**gents

Recombinant human TGF-β1 (580704) was obtained from Biolegend (San Diego, CA). CQ (C6628), 3-MA (M9281) and Baf a1 (B1793) were all purchased from Sigma-Aldrich (St. Louis, MO). The anti-LC3B antibody (L7543) was obtained from Sigma-Aldrich. Oil Red O (O9755) was purchased from Sigma-Aldrich. BODIPY 558/564 Red C12 (D3835) and BODIPY 493/503 (D3922) were both obtained from Invitrogen. DAPI (D9542) was obtained from Sigma-Aldrich, and Alexa Flour 488 or 594-conjugated anti-mouse or anti-rabbit IgG were purchased from Jackson ImmunoResearch Laboratories (West Grove, PA). The anti-Beclin-1 antibody (NBP1-00085) was purchased from Novus Biologicals (Littleton, CO). Anti-LAMP1 (ab24170), anti-SQSTM1 (ab56416) and anti- α-SMA (ab124964) antibodies were all obtained from Abcam (Cambridge, MA). Anti-cleaved caspase-3 antibody (#9664) was obtained from Cell signaling Technology (Danvers, MA), and anti-GAPDH antibody (sc-365062) was purchased from Santa Cruz (Santa Cruz, CA).

### Immunofluorescence (IF)

For kidney tissues, paraffin-embedded mouse kidneys were sectioned (4 μm), de-paraffinized and subsequently blocked in PBS containing normal donkey serum (10%) (Vector laboratories) for 30 min. Antigen retrieval was performed using citrate buffer (0.01 M, pH 6.0) prior to incubation with primary antibodies. The cells were pre-cultured on coverslips, followed by treatment as indicated, subsequent fixation with paraformaldehyde (4%) and blocking with PBS containing normal donkey serum (10%) prior to incubation with primary antibodies. The next day, the samples were incubated with Alexa Flour-conjugated secondary antibodies, DAPI or BODIPY for 1 h. Images were collected using confocal laser microscopy (Olympus FV1200, Tokyo, Japan). Exposure settings were unchanged throughout acquisition. Staining was analyzed using ImageJ software (National Institutes of Health, Bethesda, MD) and for BODIPY staining, at least one hundred cells of each group were analyzed.

### Oil Red O staining

Frozen sections (5 μm) of kidney tissues or cell coverslips were first washed in PBS and subsequently fixed with paraformaldehyde (4%) for 30 min. The samples were incubated in 60% (vol/vol) isopropyl alcohol for 3 min, followed by staining with freshly prepared 60% Oil Red O (100% solution: 0.5 g of Oil Red O dissolved in 100 ml of isopropylene) for 30 min, 60% (vol/vol) isopropyl alcohol for 1 min and washing with PBS. The samples were counterstained with hematoxylin. The slides were visualized using an Olympus microscope (Olympus, Tokyo, Japan). LDs number and LDs size were quantified using Image J software following standard protocols^48^ (For Oil Red O staining, at least one hundred cells of each group were analyzed).

### Electron microscopy

Kidney cortices were fixed in paraformaldehyde (4%) and glutaraldehyde (1%), dehydrated, sliced on a vibratome and recut on a microtome (40 nm) and stained with uranyl acetate overnight. The sections were analyzed using the Hitachi H600 transmission electron microscope (Hitachi, Tokyo, Japan).

### Western blotting

Kidney tissues or cell samples were lysed in RIPA lysis buffer (containing 1%PMSF and 1% protease inhibitor cocktail (all from Sigma-Aldrich)). Protein concentrations were measured by BCA assay (Pierce). Samples were loaded into the wells of SDS-PAGE gel with equal amounts and subjected to electrophoresis in a XCell SureLock mini-cell system (Invitrogen). After electrophoresis, samples were then transferred to PVDF membranes and blocked with 5% milk. The membranes were then incubated with indicated primary antibodies and following secondary IRDye 800CW labeled antibodies (LI-COR Biotechnology, Lincoln, NE). Odyssey CLx infrared imaging system (LI-COR Biotechnology) were used to scan the membranes and Quantity One analysis software (Bio-Rad Laboratories, Hercules, CA) was applied to quantify the band intensity of blotted proteins.

### Flow cytometry

For intracellular staining of Red C12 fluorescence, HK-2 cells were first collected, fixed and permeabilized with Cytofix/Cytoperm and Perm/Wash buffers (BD Biosciences) according to the manufacturer’s instructions. FACS was detected with Accuri C6 cytometer (BD Biosciences), the data were calculated using FlowJo software (Tree Star, Ashland, OR).

### BODIPY 558/564 Red C12 pulse-chase assay

The BODIPY 558/564 Red C12 pulse-chase assay was performed as previously described^29^. In this experiment, HK-2 cells cultured in DMEM/F12 supplemented with 10% FBS containing 1 μM BODIPY 558/564 Red C12 were chased for 16 h in the absence or presence of TGF-β1 (50 ng/ml) for 24 h. To label the LDs, BODIPY 493/503 (40 ng/ml) was added for 1 h. Images were collected using confocal laser microscopy (Olympus FV1200), and colocalization was quantified using Image J analysis software.

### Histological analysis

Kidney tissues were fixed with 4% paraformaldehyde prior to embedding in paraffin. Paraffin sections (4 μm) were used for HE, Masson Trichrome staining (MTS) or immunohistochemistry staining (IHC) according to the manufacturer’s instructions. The samples were examined using an Olympus microscope, and the images were analyzed using Image J software (For HE and IHC staining, twenty images (×200) of each group from three independent experiments were analyzed).

### TUNEL staining

The TdT-mediated dUTP nick end labeling (TUNEL) was performed using the in situ Cell Death Detection Kit (Roche, Indianapolis, IN) according to the manufacturer’s instructions. The tissue sections were detected using confocal laser microscopy (Olympus FV1200, Tokyo, Japan), and the images were analyzed using Image J software.

### Statistics

All the data are presented as the means ± SEM. Statistical analysis was performed using GraphPad Prism (Version. 7.0a) software. For data analyses with one variable, one-way ANOVA multiple comparison was used, and 2-tailed unpaired or paired Student’s *t* test analysis was used for two-group comparisons. Dunnett’s test was conducted if more than two groups were compared. For correlation plots, Pearson’s correlations were applied. *P* values < 0.05 were considered statistically significant.

## Electronic supplementary material

Supplementary Data
