# Supplementary material for: Autophagy activation contributes to lipid accumulation in tubular epithelial cells during kidney fibrosis
Source: Cell Death Discov. 2018 Jun 27;4:39. doi: 10.1038/s41420-018-0065-2 (PMC6060103; doi:10.1038/s41420-018-0065-2)
Supplement: Supplementary file 1 — Supplementary Data [file 41420_2018_65_MOESM1_ESM.docx]

**Supplementary Data**

**Figure legend**

**Figure S1. BECN1 is down-regulated in UUO-induced fibrotic kidney, as well as in TGF-β1-treated HK-2 cells.** (a) Kidney samples were collected for western blotting staining with Beclin-1 antibodies. GAPDH sets as loading control. Schematic representation of band intensity of indicated proteins. Data are calculated from three independent experiments and are expressed as the mean ± SEM. * P <0.05. (a) Cell lysate were collected for western blotting staining with Beclin-1 antibodies. GAPDH sets as loading control. Schematic representation of band intensity of indicated proteins. Data are calculated from three independent experiments and are expressed as the mean ± SEM. * P <0.05.
